# Temporal scaffolding and agentic pathways: a mixed-methods analysis of hope as a buffer in the personality–stress relationship

**DOI:** 10.3389/fpsyg.2026.1808290

**Published:** 2026-06-30

**Authors:** Lizao Chen, Liang Yin

**Affiliations:** 1 Institute of Dunhuang Studies, Lanzhou University, Lanzhou, Gansu, China; 2 International Education College, Harbin University of Commerce, Harbin, China

**Keywords:** Chinese adults, hope, mixed-methods research, moderated mediation, neuroticism, perceived stress, self-esteem, thematic analysis

## Abstract

**Introduction:**

This study examined hope as a buffer in the process linking personality to perceived stress via self-esteem.

**Methods:**

Using an explanatory sequential mixed-methods design (QUAN → qual), 425 Chinese adults completed measures of neuroticism, conscientiousness, self-esteem, hope, and perceived stress. Quantitative analysis employed latent moderated mediation modeling. Follow-up interviews were conducted with 20 purposefully selected participants (low self-esteem, high/low hope).

**Results:**

Quantitative analysis confirmed that self-esteem mediated the personality–stress relationship, and this mediation was moderated by hope (*β* = 0.16). The negative association between self-esteem and stress was significantly weaker for individuals with high hope, and the detrimental indirect effect of neuroticism was attenuated by 45% compared to those with low hope. Interviews revealed three buffering mechanisms: Temporal Scaffolding, Self-Worth Decoupling, and Agentic Pathway Generation.

**Discussion:**

The findings demonstrate that hope acts as an active psychological resource, providing specific cognitive and behavioral strategies that mitigate the impact of low self-esteem on stress, offering nuanced insights for targeted interventions.

## 1 Introduction

Perceived stress, the global appraisal of life as unpredictable and overwhelming (Cohen et al., 1983), is a critical health determinant. Its origins lie not merely in external events but in the transactional process of appraising and coping with demands (Lazarus and Folkman, 1984). Within this process, stable personality traits shape threat perception and resource evaluation. Neuroticism, a core Five-Factor Model trait marked by negative affectivity and emotional instability (McCrae and Costa, 2008), is a potent vulnerability factor and the strongest personality predictor of perceived stress (Piekarska, 2020). It is important to clarify that the language of “effect” and “pathway” used throughout this manuscript reflects theoretical and statistical assumptions derived from the transactional model and prior longitudinal evidence, not causal claims from our cross-sectional data. Several large-scale longitudinal studies have shown that neuroticism predicts subsequent increases in perceived stress over time (e.g., Kessler et al., 2009), and conscientiousness predicts lower perceived stress in prospective designs (Ebstrup et al., 2011). Meta-analyses of cross-lagged panel studies further support that personality traits antecede stress appraisals rather than merely reflecting concurrent distress (Anglim et al., 2020; Lüdtke et al., 2018). This vulnerability operates indirectly; neuroticism erodes self-worth through negative affectivity and self-criticism (Judge et al., 2002; Robins et al., 2001), and depleted self-esteem then amplifies stress appraisals by fostering helplessness (Bandura, 1997). While this mediated pathway is well-supported, a key question remains: what resources can buffer this sequence?

Hope, a future-oriented motivational state comprising goal-directed agency and pathways thinking (Snyder et al., 1991), is a promising candidate. A cornerstone of positive psychology (Seligman and Csikszentmihalyi, 2000), hope is distinguished from passive optimism by its active focus on agency and planning (Bryant and Cvengros, 2004; Snyder, 2002). It is a well-documented resource for resilience and wellbeing (Rand and Cheavens, 2009), with a strong inverse relationship to distress (Alarcon et al., 2013). However, research has primarily examined hope as a direct predictor or mediator. Its potential role as a conditional factor that moderates established vulnerability pathways—specifically, the link between low self-esteem and perceived stress within a mediation model—remains untested. This is a significant gap, as understanding the conditions under which the self-esteem-stress link is most potent is vital for targeted interventions (Hayes, 2018; Preacher et al., 2007).

This study therefore proposes and tests an integrated moderated mediation model to investigate if hope buffers the indirect effect of core personality traits on perceived stress via self-esteem. We focus on neuroticism (vulnerability) and conscientiousness (protection) in a sample of Chinese adults—a population navigating rapid socioeconomic change where identifying buffers is a priority (Liu et al., 2014). We hypothesize the mediation pathway will be conditional on hope, with higher hope attenuating the negative association between low self-esteem and high perceived stress. To comprehensively explain this process, we employ an explanatory sequential mixed-methods design (Creswell and Plano Clark, 2018). The quantitative phase tests the model using latent variable modeling. The qualitative phase then explores the lived experience of individuals with low self-esteem but varying hope to uncover the specific mechanisms behind the buffering effect. This integration moves beyond identifying a statistical interaction to explicate how hope functions as an active resource within a core vulnerability process.

## 2 Literature review

We organize the theoretical background around a single integrative framework: the transactional model of stress and coping (Lazarus and Folkman, 1984) extended by Conservation of Resources (COR) theory (Hobfoll, 1989, 2002). In this view, perceived stress arises from a mismatch between situational demands and perceived coping resources. Personality traits (e.g., neuroticism, conscientiousness) shape both threat appraisals and the accumulation or depletion of key resources such as self-esteem. Hope then functions as an additional resource that can alter the appraisal process. Rather than treating each theory separately, we use this unified lens to explain (a) why self-esteem mediates the personality–stress link, (b) why low self-esteem amplifies stress, and (c) how hope buffers that amplification. The following subsections develop each part of this integrated argument.

### 2.1 The personality-self-esteem-stress pathway

To further clarify, perceived stress is distinct from both the presence of objective stressors and the physiological stress response. Objective stressors are measurable events (e.g., work deadlines, relationship conflicts), but two individuals exposed to the same stressor can report very different levels of perceived stress depending on their appraisal and coping resources (Lazarus and Folkman, 1984). The Perceived Stress Scale (PSS-10) used in this study captures this subjective appraisal by asking how often in the past month respondents felt that difficulties were piling up so high they could not overcome them—a direct measure of secondary appraisal (Cohen et al., 1983). Therefore, in this study we focus on perceived stress as the proximal outcome, consistent with our transactional framework.

It is widely accepted that subjective appraisal processes play a central role in shaping psychological distress (Cohen et al., 1983; Lazarus and Folkman, 1984). However, the terms “stress” and “distress” are often used interchangeably, which can obscure important distinctions. In this manuscript, we follow the transactional model’s terminology: *stress* refers to the cognitive appraisal of a situation as exceeding one’s resources (i.e., perceived stress), whereas *distress* denotes the negative emotional state (e.g., anxiety, sadness, irritation) that may result from such appraisals (Lazarus, 1991; Monroe and Slavich, 2016). Our primary outcome, perceived stress (measured by the PSS-10), captures the appraisal component, not the emotional response per se. Nevertheless, the two are closely linked, and we occasionally refer to “distress” when discussing broader psychological outcomes (e.g., Alarcon et al., 2013). The transactional model of stress frames it as a dynamic person-environment interaction, not merely an external event (Lazarus and Folkman, 1984). This model, building on earlier foundations (Lazarus, 1966), involves primary appraisal (evaluating threat) and secondary appraisal (assessing coping resources) (Lazarus, 1991). Perceived stress—the global sense that life is unpredictable and overwhelming (Cohen et al., 1983)—is therefore shaped by stable personality traits (Folkman and Moskowitz, 2004). The mechanisms through which personality traits influence perceived stress include (a) appraisal tendencies (e.g., neurotic individuals perceive more threat in ambiguous situations), (b) differential exposure to stressors (e.g., neuroticism predicts more frequent interpersonal conflicts), and (c) coping styles (e.g., conscientiousness promotes active problem-solving whereas neuroticism favors avoidance or rumination) (Bolger and Zuckerman, 1995; Connor-Smith and Flachsbart, 2007; Suls and Martin, 2005). Within our integrative framework, personality traits influence stress primarily by affecting the availability of a core psychological resource: self-esteem.

Before proceeding, we clarify the terms self-esteem and self-worth, as the literature sometimes uses them interchangeably. Self-esteem is formally defined as a person’s overall subjective evaluation of their own worth (Rosenberg, 1965). Self-worth, in contrast, refers to the basic, often unconditional sense of being valuable as a person, which can be seen as the core content of self-esteem. In practice, the Rosenberg Self-Esteem Scale (RSES) operationalizes self-esteem through items that directly ask about satisfaction with oneself and feelings of worth (“I feel that I am a person of worth”). Thus, while self-worth is the underlying judgment, self-esteem is the broader construct that encompasses that judgment along with its affective and cognitive components. In this manuscript, we follow Rosenberg’s tradition and use self-esteem as the inclusive term for global self-evaluation, and we use “self-worth” only as a synonym for the evaluative content of self-esteem, not as a separate construct. This is consistent with how the RSES is scored and interpreted.

Personality traits strongly shape these appraisals. Neuroticism, a core Five-Factor Model trait rooted in lexical tradition (Goldberg, 1993), involves a stable tendency toward negative affect and emotional instability (McCrae and Costa, 2008). It is a key vulnerability factor in diathesis-stress models (DeNeve and Cooper, 1998; Lahey, 2009), with links to maladaptive stress responses noted early in trait theory (Eysenck, 1967). Individuals high in neuroticism show cognitive reactivity, appraising ambiguity as threatening, and tending to ruminate and engage in catastrophic thinking (Bolger and Zuckerman, 1995; Nolen-Hoeksema, 2000; Suls and Martin, 2005). This pattern affects both primary and secondary appraisal, heightening threat perception and undermining coping confidence (Gunthert et al., 1999), making neuroticism the strongest personality predictor of perceived stress (Piekarska, 2020). Some evidence suggests neuroticism may be a common cause of both low self-esteem and distress (Mu et al., 2019). Crucially, from a resource perspective, neuroticism depletes self-esteem.

The neuroticism-stress link is critically mediated by self-esteem. Following the clarification above, self-esteem is defined as overall self-evaluation (Rosenberg, 1965) and has long been viewed as a mediator between personality traits and outcomes (Coopersmith, 1967). Neuroticism, characterized by self-criticism, erodes this resource (Judge et al., 2002; Robins et al., 2001), especially when self-esteem is already low (Oropesa Ruiz et al., 2022). Within our integrative framework, the transactional model of stress explains why depleted self-worth increases stress susceptibility: low self-esteem weakens secondary appraisal, fostering a sense of helplessness (Bandura, 1997). At the same time, Conservation of Resources (COR) theory frames low self-esteem as a loss of a personal resource, which amplifies perceived stress and can trigger further resource loss spirals (Hobfoll, 2002). Complementing these, sociometer theory (Leary and Baumeister, 2000) adds that low self-esteem signals social threat and rejection, heightening sensitivity to evaluation and thereby increasing stress. Vulnerability is influenced not only by the level but also by the stability and contingencies of self-esteem (Crocker and Wolfe, 2001; Kernis, 2003). Longitudinal work shows that self-esteem lability following negative events links adversity to distress, particularly for neurotic individuals (Auerbach et al., 2010), and traits like neuroticism predict self-esteem trajectories after setbacks (Schellenberg et al., 2025). Thus, the three perspectives converge: the transactional model describes the appraisal process, COR theory explains resource dynamics, and sociometer theory highlights social signaling—all identifying self-esteem as the central mediator.

This mediating role extends beyond neuroticism. Self-esteem accounts for the influence of other Big Five traits (e.g., agreeableness, conscientiousness, openness) on outcomes such as depressive symptoms, technostress, creativity, and body esteem (Hong et al., 2020; Zafar et al., 2020; Shi et al., 2015; Skorek et al., 2014). In addition, self-esteem mediates the relationship between personality traits and relationship satisfaction (Hill et al., 2016). Related cognitive resources, like general self-efficacy, also mediate the personality-stress link (Ebstrup et al., 2011; Şahin and Çetin, 2017). In applied settings, self-esteem and perceived stress together mediate the impact of work stressors on depression (Lee et al., 2013), and self-esteem can sometimes buffer neuroticism’s link to maladaptive coping (Amestoy et al., 2023). Collectively, this evidence supports a robust model where personality, especially neuroticism, undermines self-esteem, which in turn amplifies stress. This established pathway raises the pivotal question that our framework next addresses: what personal resources can buffer this cascade? Hope, as a cognitive-motivational resource, is the candidate we turn to.

### 2.2 Hope as a key psychological resource

Hope is a well-established psychological resource. While the positive psychology movement formalized the study of human strengths (Seligman and Csikszentmihalyi, 2000), interest in hope’s role in mental health and motivation has earlier roots (Menninger, 1959; Stotland, 1969). The dominant cognitive theory defines hope as a future-oriented, goal-directed state comprising pathways thinking (generating routes to goals) and agency thinking (the motivation to pursue them) (Snyder, 2000, 2002; Snyder et al., 1991). This conceptualization builds on Stotland's (1969) view of hope as goal-attainment expectation, framing it as an active, cognitive process distinct from passive optimism. In the present study, hope is operationalized using the Adult Hope Scale (Snyder et al., 1991), which captures both pathways and agency; a total score serves as the moderator, reflecting the overall strength of goal-directed thinking.

It is crucial to distinguish hope from related constructs. Dispositional optimism is a generalized expectation for positive outcomes (Scheier and Carver, 1985), whereas hope centers on one’s perceived capacity to devise and execute plans (Magaletta and Oliver, 1999)—a “positive motivational state” of agency and pathways (Snyder et al., 1991, p. 287). Hope is often a superior predictor of performance outcomes due to this agentic foundation (Bryant and Cvengros, 2004). Hope also differs from self-efficacy. Self-efficacy refers to a person’s belief in their ability to execute a specific task or behavior in a given situation (Bandura, 1997). Hope, in contrast, is broader: it involves generating multiple routes to a desired goal (pathways) and the motivation to pursue them (agency), regardless of confidence in any single specific action (Snyder, 2002). A person can have low self-efficacy for a particular task (e.g., public speaking) but still be hopeful about achieving a larger career goal by finding alternative ways (e.g., writing, small group presentations). Thus, hope is less context-bound than self-efficacy and emphasizes goal-reappraisal and flexible problem-solving rather than task-specific mastery.

Hope also overlaps with but is distinct from resilience. Resilience is broadly defined as the ability to bounce back or adapt successfully in the face of adversity (Luthar et al., 2000; Kalisch et al., 2015). However, resilience is often treated as a broad outcome or a set of protective factors, whereas hope is a specific cognitive-motivational process. Cognitive resilience refers to the capacity to maintain or regain flexible thinking under stress (Parsons et al., 2016). Hope contributes to cognitive resilience because pathways thinking generates alternative solutions, preventing the cognitive constriction that low self-esteem can cause. Emotional resilience involves regulating negative emotions and recovering from affective distress (Tugade and Fredrickson, 2004). Hope fosters emotional resilience by redirecting attention from present failure to future possibilities (agency), which buffers the emotional impact of setbacks. Unlike general resilience models that aggregate many protective factors (e.g., optimism, self-regulation, social support), hope offers a parsimonious, theory-driven construct with two well-defined components that can be directly measured and trained. In the present study, we focus on hope as a specific moderator because its active, goal-directed nature directly counteracts the helplessness and cognitive constriction produced by low self-esteem—a more targeted mechanism than global resilience would provide. It is recognized as a core character strength (Peterson and Seligman, 2004).

Empirical evidence solidifies hope’s role as a transdiagnostic asset for wellbeing. Meta-analytic work confirms its strong inverse relationship with psychopathology (Alarcon et al., 2013). Hope predicts positive functioning across mental and physical health, academics, and relationships (Rand and Cheavens, 2009), aiding illness adjustment and treatment adherence (Griggs and Walker, 2016; Snyder, 2000). These benefits stem from documented mechanisms: high-hope individuals are better problem-solvers, appraise stressors as challenges, show greater cognitive flexibility, and mobilize support effectively (Colla et al., 2022; Snyder et al., 1996), consistent with its core definition (Snyder et al., 1991).

Hope’s positive impact is evident across diverse populations and contexts. It predicts wellbeing in groups from ethnic minority adolescents to Chinese young adults (Vacek et al., 2010; Zhang et al., 2025). Its protective role is pronounced during adversity; for example, it predicted life satisfaction during the COVID-19 pandemic by bolstering self-esteem (Chan and Huang, 2024). In trauma recovery, hope is a key mechanism linking social support to post-traumatic growth, sometimes sequentially with self-esteem (Zhou et al., 2018). Hope is also studied within broader resource constellations, like psychological capital, where it mediates links between personality and adaptive outcomes such as creativity (Hong et al., 2020). This extensive support, however, primarily treats hope as a direct predictor or a mediator. Within our transactional-resource framework, hope is best conceptualized as a “personal resource” (Hobfoll, 2002) that operates at the secondary appraisal stage: it does not raise self-esteem directly, but it changes how a person responds to low self-esteem when facing a stressor. The question of whether hope can function as a moderator—specifically, whether it can alter the strength of the relationship between low self-esteem and perceived stress—requires a closer look at the deficits that low self-esteem creates. Low self-esteem typically narrows a person’s perceived coping options and fosters a sense of helplessness (Bandura, 1997; Kernis, 2003). Hope’s pathways thinking directly counteracts this constriction by keeping alternative routes mentally available, even when one doubts one’s own worth. Likewise, hope’s agency thinking supplies the motivational push to try those routes, preventing the passive resignation that would otherwise turn low self-esteem into high perceived stress. Thus, hope is not merely another resource that lowers stress directly; it is a conditional buffer that weakens the otherwise tight link between feeling inadequate and feeling overwhelmed. This reasoning integrates Conservation of Resources theory (Hobfoll, 2002), where hope interrupts loss spirals triggered by low self-worth, with the transactional model (Lazarus and Folkman, 1984), where hope alters secondary appraisal. Preliminary evidence from other contexts supports such buffering (Jiang et al., 2025; Zhang et al., 2025), but no study has tested whether hope moderates the mediated pathway from personality to perceived stress via self-esteem—a gap the current study addresses. This integrated perspective positions hope as a potent psychological resource capable of moderating vulnerability, which directly leads to our moderated mediation hypothesis (H1).

### 2.3 The buffering hypothesis: hope as a moderator of vulnerability

The established vulnerability pathway from personality to stress via self-esteem raises the question of whether psychological resources can buffer or attenuate this process. Consistent with our integrative framework, the classic stress-buffering hypothesis proposes that resources can mitigate adversity’s impact (Cohen and Wills, 1985), an idea consistent with earlier interactional models (French et al., 1974). Conservation of Resources (COR) Theory extends this by defining stress as a reaction to threatened or lost resources (Hobfoll, 1989, 2002). Within this combined view, hope is a key “personal characteristic resource.” Its active function theoretically facilitates acquiring and protecting other resources (e.g., self-esteem) and alters threat appraisals (Lazarus and Folkman, 1984). We therefore hypothesize that hope acts as a cognitive-motivational buffer, moderating the low self-esteem–perceived stress link—a test aligning with principles for examining conditional effects (Baron and Kenny, 1986).

The buffering mechanism is rooted in hope’s two-component structure, which counteracts the specific deficits created by low self-esteem. Hope theory originally posited that agency and pathways are essential for overcoming obstacles (Snyder et al., 1991). Low self-esteem often fosters helplessness and cognitive constriction (Bandura, 1997). Hope’s pathways thinking promotes cognitive flexibility and problem-solving (Snyder et al., 1996), which aligns with broaden-and-build theory (Fredrickson, 2001) as a supplementary mechanism: positive emotions from hope broaden thought-action repertoires, helping to prevent COR’s “loss spirals” (Hobfoll, 2002). Concurrently, agency thinking sustains effort and approach-oriented coping (Carver et al., 1989), contrasting with avoidance linked to low self-worth. Thus, within our framework, hope operates at two points: (1) during secondary appraisal, by increasing perceived pathways (transactional model), and (2) by preventing resource depletion (COR theory).

Emerging research supports hope’s moderating role in stress processes, though not in the specific integrated model tested here. Hope buffers the impact of interpersonal stress on depression in adolescents (Jiang et al., 2025) and moderates pathways between meaning in life, stress, and wellbeing (Zhang et al., 2025), mirroring findings for optimism (Rikkers and Lawrence, 2021). Hope and self-esteem can operate synergistically, acting as sequential mediators in post-trauma growth (Zhou et al., 2018). The role of resources is context-dependent; for example, conscientiousness may correlate with higher stress in technology-saturated workplaces (Zafar et al., 2020), underscoring the need to examine conditional processes.

While hope’s benefits (Alarcon et al., 2013; Valle et al., 2006) and the buffering effects of resources like social support (Cohen and Wills, 1985) and optimism (Scheier et al., 2001) are documented, a gap remains. Although conceptual groundwork for testing moderated mediation exists (James and Brett, 1984), hope’s specific function as a moderator of the mediated pathway from core personality traits (neuroticism, conscientiousness) to stress via self-esteem is untested. This study addresses that gap by investigating a moderated mediation model to examine how hope conditionally alters this vulnerability process—a direct test of the buffering hypothesis within our integrative framework.

### 2.4 Cultural considerations: transcultural relevance of core constructs

The present study was conducted in mainland China, a cultural context that differs from the Western settings where many psychological constructs were originally developed. We therefore examine the cultural specificity and generalizability of each core concept—perceived stress, self-esteem, hope, resilience, and optimism—drawing on cross-cultural research and validation studies in Chinese populations.

Perceived stress, as measured by the PSS-10, has been validated in Chinese samples, with studies supporting its reliability and factor structure (Wang et al., 2011). The experience of feeling overwhelmed by demands is universal, but Chinese adults may report higher perceived stress due to rapid socioeconomic change and collectivist pressures to maintain social harmony (Liu et al., 2014). In our sample, the mean PSS-10 score (*M* = 2.33) was comparable to other urban Chinese studies, supporting its relevance.

Self-esteem, traditionally studied in individualistic cultures, has been critiqued as less central in collectivist societies where self-worth derives more from role fulfillment and group belonging (Heine et al., 1999). However, a large-scale meta-analysis shows that the Rosenberg Self-Esteem Scale performs similarly across nations, with acceptable reliability and convergent validity in China (Schmitt and Allik, 2005). Chinese self-esteem tends to be lower on average than in Western samples, but its functional role—mediating personality and distress—appears equivalent (Schmitt and Allik, 2005). Our results (*α* = 0.90) confirm good reliability, and the mediation pattern aligns with prior Chinese studies (Shi et al., 2015).

Hope, as defined by Snyder’s cognitive theory, has been validated in Chinese samples using the Adult Hope Scale, showing a two-factor structure similar to Western samples (Chen et al., 2009). Cultural nuances exist: Chinese conceptions of hope may emphasize collective goals (e.g., family prosperity) more than individual achievement (Bernardo, 2010). The pathways and agency components remain relevant, but the content of goals may differ. Our qualitative themes (Temporal Scaffolding, Self-Worth Decoupling) emerged from Chinese participants’ narratives, suggesting that hope operates through culturally adapted mechanisms. Future cross-cultural comparisons are needed to test whether these mechanisms generalize.

Resilience, broadly defined as successful adaptation despite adversity, has been studied in China using both Western scales (e.g., Connor-Davidson Resilience Scale) and indigenous measures (Yu and Zhang, 2007). Chinese resilience often incorporates family support and interpersonal harmony more prominently than individual traits, reflecting collectivist values (Bernardo et al., 2017). Our focus on hope as a specific cognitive resource rather than global resilience is consistent with the need for culturally nuanced constructs; hope’s pathways and agency may be less confounded with relational factors.

Optimism, distinguished from hope in Section 2.2, has been shown to be similarly beneficial in China, with a Chinese version of the Life Orientation Test (LOT-R) exhibiting adequate reliability and validity (Lai and Yue, 2000). However, Chinese optimism may be more cautious and less self-enhancing than Western optimism (Chang and Sanna, 2001). In our study, we prioritize hope over optimism because hope’s agentic, plan-focused nature is more directly amenable to intervention and less influenced by cultural differences in self-positivity.

Overall, while each construct shows good psychometric performance in Chinese samples, their expression and relative importance may be shaped by collectivist values, rapid social change, and Confucian heritage. Our findings should be interpreted with these cultural specificities in mind, and future research should directly compare buffering mechanisms across cultures to establish transcultural validity.

### 2.5 The present study: gaps and hypotheses

The literature supports two primary models: a vulnerability pathway where personality erodes self-esteem and increases stress (Judge et al., 2002; Lazarus and Folkman, 1984), situated within diathesis-stress frameworks (Monroe and Simons, 1991), and a resilience model where hope promotes wellbeing (Snyder, 2002). These models remain inadequately integrated. Self-esteem is a well-established mediator, translating Big Five traits into outcomes like depression, relationship satisfaction, and body esteem (Lee et al., 2013; Shi et al., 2015; Skorek et al., 2014; Hill et al., 2016), just as other agentic resources like self-efficacy do for stress (Ebstrup et al., 2011; Şahin and Çetin, 2017).

Concurrently, hope’s protective role is robust, evidenced across contexts including trauma and public health crises (Chan and Huang, 2024; Zhou et al., 2018). Emerging research indicates it can function as a moderator, buffering the impact of interpersonal stress (Jiang et al., 2025) and conditioning other mediated pathways (Zhang et al., 2025). Hope and self-esteem also show synergistic relationships, operating as sequential mediators (Zhou et al., 2018) or with self-esteem mediating hope’s link to wellbeing (Chan and Huang, 2024). A critical gap persists, however. While self-esteem mediates the personality-stress link and hope moderates stress outcomes, no study has tested if hope moderates the mediating pathway itself—specifically, whether it buffers the low self-esteem-stress link within a model originating from core personality traits. This tests a specific moderated mediation model for which analytical frameworks exist (Hayes, 2015; Preacher et al., 2007).

Why a moderated mediation model rather than a sequential mediation model? A sequential mediation model would position hope as an additional mediator that comes before or after self-esteem in a causal chain (e.g., personality → self-esteem → hope → stress, or personality → hope → self-esteem → stress). However, our theoretical framework rules out these sequences. Hope does not arise from low self-esteem; rather, hope is a relatively stable personal resource that is only moderately correlated with self-esteem (*r* = 0.55 in our sample). Likewise, hope is not a direct outcome of personality in a way that would then affect self-esteem. Instead, theory and prior evidence suggest that hope operates as a conditional buffer: it changes the strength of the relationship between low self-esteem and perceived stress, without being caused by low self-esteem or serving as an intervening variable. Moderated mediation is therefore the appropriate specification because we hypothesize that the indirect effect of personality on stress via self-esteem varies depending on the level of hope. A sequential mediation model would incorrectly imply that hope transmits the effect of personality (or self-esteem) rather than altering it.

Selection of neuroticism and conscientiousness (excluding other Big Five traits). We focus on neuroticism as the primary vulnerability trait because meta-analytic evidence identifies it as the strongest personality predictor of perceived stress (Piekarska, 2020) and because its link to self-esteem erosion is well-documented (Judge et al., 2002). We include conscientiousness as a contrasting protective trait for two reasons. First, conscientiousness is consistently associated with higher self-esteem and lower perceived stress (McCrae and Costa, 2008; Shi et al., 2015). Second, and more critically for our model, conscientiousness shares conceptual overlap with hope: both involve goal-directed persistence and planful action. This overlap allows us to test a substitution hypothesis—that hope may render the protective effect of conscientiousness less necessary. We exclude agreeableness, openness, and extraversion because prior meta-analyses show weaker or inconsistent direct associations with perceived stress via self-esteem (Alarcon et al., 2013; Piekarska, 2020). Including them would increase model complexity without clear theoretical grounding for moderation by hope. Our focus on two theoretically contrasting traits (vulnerability vs. protection) keeps the model parsimonious and testable, consistent with recommendations for moderated mediation research (Hayes, 2018).

Identifying for whom and when this vulnerability pathway is strongest is crucial for targeted intervention (Hayes, 2018; Preacher et al., 2007). This study addresses that gap by testing an integrated moderated mediation model within a sample of Chinese adults, a population facing significant socioeconomic stress where identifying buffers is *a priori*ty (Liu et al., 2014). While self-esteem and hope are moderately correlated, they are conceptually distinct: self-esteem represents an evaluative judgment of one’s current worth, whereas hope is a cognitive-motivational construct focused on the agentic generation of pathways toward future goals (Snyder, 2002). We argue that hope provides a unique regulatory mechanism that self-esteem alone does not. We hypothesize hope will alter the strength of the mediational pathway, leading to our primary hypothesis:

*H1*: The indirect effect of personality on perceived stress through self-esteem will be conditional on hope. Specifically, hope will buffer the detrimental indirect effect of neuroticism (attenuation). For conscientiousness, we expect a substitution effect, where the protective indirect effect of conscientiousness is less pronounced at higher levels of hope due to the overlapping resource of high agentic thinking.

We test this using an explanatory sequential mixed-methods design (Creswell and Plano Clark, 2018), a recognized approach for explaining quantitative results qualitatively (Ivankova et al., 2006). The quantitative phase employs latent moderated structural equation modeling (Kline, 2015) to test the conditional effect. The subsequent qualitative phase then explicates the lived experience behind the statistical interaction (Teddlie and Tashakkori, 2009) by interviewing individuals with low self-esteem but high hope, uncovering the mechanisms of the buffering process to move beyond statistical prediction to a holistic understanding.

## 3 Methods

This study employed a two-phase, explanatory sequential mixed-methods design (QUAN → qual). This approach was strategically chosen to first test a complex moderated mediation model of perceived stress with a sample of 425 Chinese adults recruited using a stratified quota sampling strategy. The quantitative (QUAN) phase allowed us to determine *if* and *to what extent* hope buffers the deleterious relationship between low self-esteem and stress. The subsequent qualitative (qual) phase was then designed to explore *how* and *why* this buffering effect occurs, providing rich, contextualized narratives to explain the statistical interactions. This integrated design provides both statistical generalizability and deep explanatory insight. The study was conducted in accordance with the ethical principles of the Declaration of Helsinki and received full approval from the university’s Institutional Review Board.

### 3.1 Participants and procedure

Data were collected from a sample of adults in mainland China across two phases between January and April 2025. In the first phase, a quantitative survey was administered via KuRunData, a major national online survey panel that maintains a pre-registered pool of over 2.6 million Chinese residents. A stratified quota sampling strategy was employed to approximate the demographic distribution of the Chinese urban adult population (aged 25–55) based on three strata: (a) gender (male/female, target 50% each), (b) age group (25–39 years and 40–55 years, target 50% each), and (c) geographic region defined by city tier (Tier 1: Beijing, Shanghai, Guangzhou, Shenzhen—target 30%; Tier 2: provincial capitals such as Chengdu, Wuhan, Hangzhou—target 40%; Tier 3: smaller prefectural cities—target 30%). Quotas were set proportionally to the most recent China Census urban population estimates. KuRunData’s algorithmic sampling system invited panelists who met the quota cells, and recruitment continued until each cell was filled within ±3% tolerance. Based on an a priori power analysis (Monte Carlo simulation for moderated mediation) requiring *N* = 400 to detect small conditional effects, we oversampled by approximately 40% to account for potential attrition and data quality filtering, initially recruiting 568 participants. After providing digital informed consent, participants completed the online survey. To ensure data integrity, two attention-check items were embedded, and respondents with completion times less than one-third of the median were excluded. Following this rigorous cleaning process, which resulted in 143 exclusions, the final quantitative sample comprised 425 adults (220 female, 51.8%; 205 male, 48.2%) with a mean age of 39.8 years (SD = 9.1). Regarding socioeconomic status (SES), participants reported their monthly household income in CNY (eight brackets from <5,000 to >50,000) and highest education level (six categories from “less than high school” to “postgraduate degree”). The sample covered the full range: 22% had monthly household income below 10,000 CNY, 48% between 10,000 and 30,000 CNY, and 30% above 30,000 CNY; 34% held a bachelor’s degree or higher. The sample thus reflected diversity in education and income levels.

Following the quantitative analysis, a second qualitative phase was conducted. To ensure the stability of the qualitative sampling frame, we employed a stratified purposeful sampling strategy specifically targeting “deviant cases” (Patton, 2015). We first identified a broad pool of eligible candidates based on quartile splits, isolating individuals in the top quartile for Hope and bottom quartile for Self-Esteem (*n* = 53). From this robust pool, we applied a stricter “intensity sampling” criterion (> 1 SD discrepancy) to select participants who most clearly exemplified the buffering phenomenon. We also screened for SES diversity; however, the final qualitative sample—recruited from the quantitative pool—skewed toward middle to high SES. Among the 20 interviewees, 17 (85%) reported monthly household income above the sample median, and 15 (75%) had at least a bachelor’s degree. This reflects the fact that individuals with higher education and income were more willing to participate in extended video interviews and had more flexible work schedules. We acknowledge this as a limitation of the qualitative component (see Section 7).

This rigorous screening identified 18 primary candidates for the Low Self-Esteem/High Hope (LSE/HH) group. Of these 18 eligible individuals, 10 (55.6% yield) agreed to participate in the interviews. Malterud’s (2016) concept of “information power” guided the sample size determination; given the highly specific selection criteria and strong theoretical background, *N* = 10 was deemed sufficient to reach saturation. A comparison group of Low Self-Esteem/Low Hope individuals (*n* = 10) was recruited using identical criteria. In total, 20 participants (12 female, 8 male) agreed to take part in these follow-up interviews. They provided separate informed consent and received a cash incentive of ¥150 (approximately €20) for their time.

### 3.2 Data collection and measures

Data were collected using online surveys (Phase 1) and remote video interviews (Phase 2). All instruments were administered in Mandarin Chinese, following a rigorous committee-based translation and back-translation procedure.

#### 3.2.1 Quantitative measures (Phase 1)

##### 3.2.1.1 Personality traits

The Chinese version of the Big Five Inventory (BFI) (John and Srivastava, 1999) was used to assess personality traits. We focused specifically on the standard 8-item Neuroticism subscale (e.g., “I am someone who worries a lot”) and the 9-item Conscientiousness subscale (e.g., “I am someone who is persistent, works until the task is finished”). Participants rated items on a 5-point Likert scale ranging from 1 (Disagree strongly) to 5 (Agree strongly).

To ensure the structural integrity of the measures within our specific cultural context, a Confirmatory Factor Analysis (CFA) was conducted on the current sample. The results supported the structural validity of the 8-item Neuroticism factor, with standardized factor loadings (*λ*) ranging from 0.68 to 0.82. Both subscales demonstrated good internal consistency and composite reliability (Neuroticism: *α* = 0.85, *ω* = 0.86; Conscientiousness: *α* = 0.84, *ω* = 0.85).

##### 3.2.1.2 Self-esteem

The 10-item Rosenberg Self-Esteem Scale (RSES) (Rosenberg, 1965) measured global self-worth (e.g., “On the whole, I am satisfied with myself”) on a 4-point scale (1 = *Strongly disagree* to 4 = *Strongly agree*). Internal consistency was excellent (*α* = 0.90, *ω* = 0.91).

##### 3.2.1.3 Hope

The 12-item Adult Hope Scale (AHS) (Snyder et al., 1991) measured goal-directed thinking (e.g., “I can think of many ways to get out of a jam”) on an 8-point scale (1 = *Definitely false* to 8 = *Definitely true*). Reliability was excellent (*α* = 0.91, *ω* = 0.91).

##### 3.2.1.4 Perceived stress

The 10-item Perceived Stress Scale (PSS-10) (Cohen et al., 1983) assessed the degree to which life has been appraised as stressful in the past month (e.g., “…how often have you felt that difficulties were piling up so high that you could not overcome them?”) on a 5-point scale (0 = *Never* to 4 = *Very often*). The scale showed good reliability (*α* = 0.87, *ω* = 0.88).

#### 3.2.2 Qualitative interviews (Phase 2)

##### 3.2.2.1 Semi-structured interviews

Data were collected through semi-structured, one-on-one interviews conducted via a secure video conferencing platform. The interview protocol was designed to explore the lived experience of coping with stress, particularly focusing on the interplay between self-esteem and hope. Sample questions included:

- *“Can you tell me about a recent situation at work or in your life that you found particularly stressful?”*
- *“During that time, how were you feeling about yourself generally? (Probing self-esteem)”*
- *“When you were in that stressful situation, what were your thoughts about the future, or about your ability to get through it? (Probing hope)”*
- *“How did those thoughts about the future help—or hinder—your ability to manage the stress you were feeling?”*

Interviews lasted 60–75 min, were audio-recorded, and transcribed verbatim.

### 3.3 Data analysis

The quantitative data were analyzed using latent moderated structural equations (LMS) in Mplus 8.8. The specified model treated Neuroticism and Conscientiousness as predictors of Self-Esteem, which was subsequently modeled as a mediator predicting the final outcome of Perceived Stress. Crucially, Hope was incorporated as a latent moderator of the path from self-esteem to perceived stress. Because LMS estimation via numerical integration does not provide standard absolute fit indices (e.g., CFI, TLI), model fit was assessed using a two-step procedure. First, we evaluated a baseline model without the interaction term using standard fit indices. Second, we compared the moderated model (*M*_1_) to the baseline model (*M*_0_) using the log-likelihood ratio test (*D*-test) and Information Criteria (AIC, BIC) to determine if the interaction significantly improved model fit. To test the significance of the conditional indirect effects—the mediating effect of self-esteem at different levels of hope—we employed bootstrapping with 10,000 resamples. Following this quantitative phase, the qualitative interview transcripts were analyzed using reflexive thematic analysis (Braun and Clarke, 2006). The analysis specifically compared the narratives between the two purposefully selected groups (Low Self-Esteem/High Hope versus Low Self-Esteem/Low Hope). The analytical process involved familiarization with the data, systematic coding, and the development and refinement of themes. To ensure rigor, two researchers independently coded a subset of transcripts to establish a consensual coding framework, and regular peer debriefing sessions were held to challenge and refine interpretations.

To integrate the findings from both phases, a mixed-methods approach was applied at two key points. First, the quantitative results directly guided the purposeful sampling strategy for the qualitative phase. Second, during the final interpretation, the emergent qualitative themes were used to explain and add depth to the statistical findings. Specifically, the themes derived from comparing the two interview groups provided a rich, narrative explanation for the buffering effect of hope observed in the quantitative model, illustrating the distinct cognitive and behavioral strategies employed by hopeful individuals facing stress in the context of low self-esteem.

## 4 Results

### 4.1 Preliminary analyses

Descriptive statistics, internal consistency estimates (Cronbach’s α and McDonald’s ω), and latent variable correlations for all primary study variables are presented in Table 1. All measures demonstrated good to excellent reliability. The correlation matrix revealed patterns consistent with our theoretical framework. Neuroticism was negatively associated with self-esteem and hope, and positively with perceived stress. Conversely, conscientiousness, self-esteem, and hope were all significantly and negatively correlated with perceived stress. These initial associations provided a strong foundation for testing the hypothesized moderated mediation model.

**Table 1:** Descriptive statistics, reliabilities, and bivariate correlations among latent variables (*N* = 425).

Variable	*M* (SD)	*α*/*ω*	1	2	3	4	5
1. Neuroticism	2.85 (0.88)	0.85/0.86	–				
2. Conscientiousness	3.91 (0.72)	0.84/0.85	−0.35^**^	–			
3. Self-esteem	2.95 (0.61)	0.90/0.91	−0.62^**^	0.41^**^	–		
4. Hope	5.44 (1.15)	0.91/0.91	−0.48^**^	0.39^**^	0.55^**^	–	
5. Perceived stress	2.33 (0.79)	0.87/0.88	0.65^**^	−0.44^**^	−0.68^**^	−0.59^**^	–

^**^p < 0.01.

### 4.2 Measurement model

Following the two-step modeling approach, we first specified and tested a comprehensive CFA measurement model. This model included all five latent variables (Neuroticism, Conscientiousness, Self-Esteem, Hope, and Perceived Stress) and their respective indicators, with all factors allowed to covary. The measurement model provided a good fit to the data, *χ*^2^(769) = 1,597.55, *p* < 0.001; CFI = 0.95; TLI = 0.94; RMSEA = 0.051 (90% CI [0.048, 0.054]); SRMR = 0.048.

Given the reliance on self-report data and the high correlation between Neuroticism and Perceived Stress (*r* = 0.65), we addressed potential Common Method Bias (CMB) and construct redundancy. First, a Harman’s single-factor test revealed that the first factor accounted for only 32.4% of the variance, well below the 50% threshold. Second, we conducted a chi-square difference test comparing our theoretical five-factor model against a four-factor model where Neuroticism and Perceived Stress were collapsed into a single latent construct. The five-factor model showed significantly superior fit (Δ*χ*^2^(4) = 412.18, *p* < 0.001), confirming that despite shared variance due to negative affectivity, they remain empirically distinct constructs. All factor loadings were statistically significant and substantial (standardized loadings ranged from 0.65 to 0.89), supporting convergent validity. Discriminant validity was also established, with the Heterotrait-Monotrait Ratio of Correlations (HTMT) values between all pairs of constructs falling below the conservative 0.85 threshold. Specifically, the HTMT value for the self-esteem and hope relationship was 0.64.

### 4.3 Structural model and moderated mediation analysis

We tested our primary hypotheses using a latent moderated structural equations (LMS) model. Prior to the inclusion of the latent interaction, the baseline model (*M*_0_) without the moderation path showed acceptable fit to the data: *χ*^2^(812) = 1715.33, *p* < 0.001; CFI = 0.94; TLI = 0.93; RMSEA = 0.052 (90% CI [0.049, 0.055]); SRMR = 0.055. To evaluate the moderated mediation model (*M*_1_), we conducted a log-likelihood ratio test. The result indicated that the inclusion of the Self-Esteem × Hope interaction significantly improved model fit, Δ − 2*LL*(1) = 13.12, *p* < 0.001. Furthermore, the Information Criteria for the interaction model (AIC = 14,210.45; BIC = 14,380.12) were lower than those of the baseline model (AIC = 14,221.57; BIC = 14,387.21), supporting the moderated structural model.

**Figure 1:**
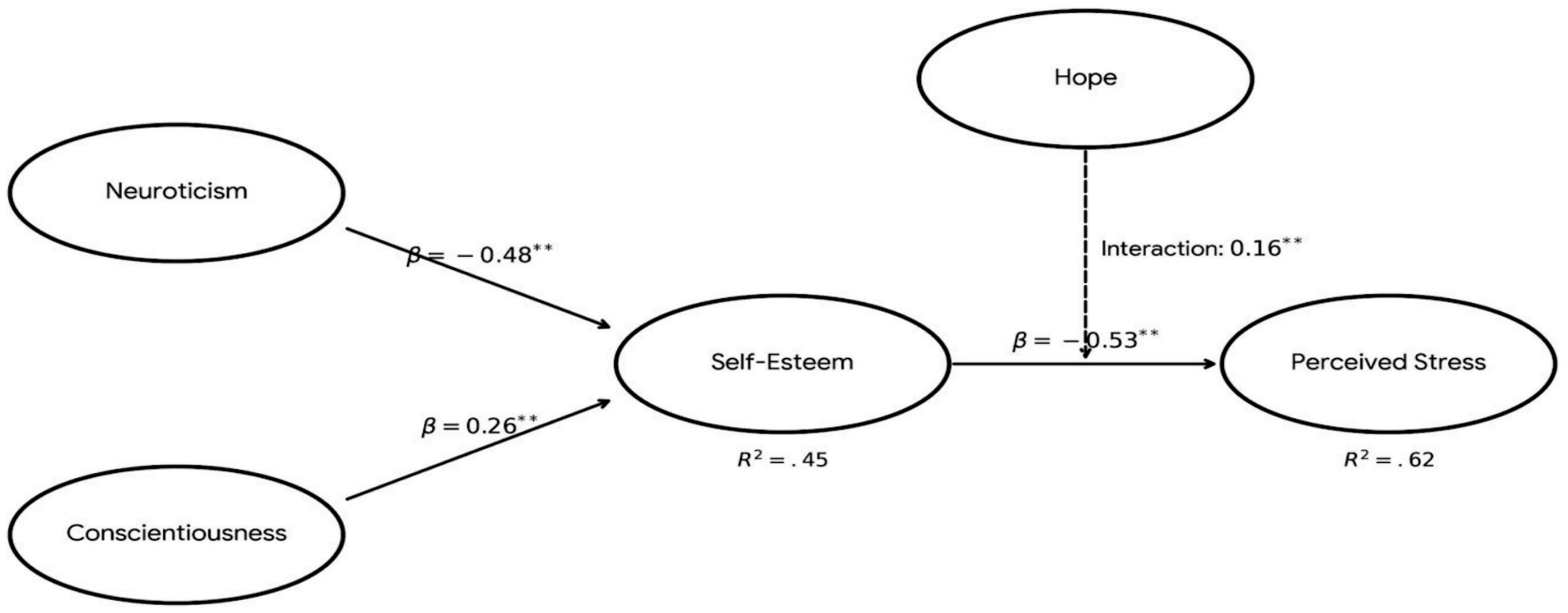
The final moderated mediation model. Standardized path coefficients (*β*) are shown for the main effects. Control variables are omitted for clarity. All displayed paths are significant at *p* < 0.01.

The model accounted for a substantial portion of the variance in the endogenous variables, explaining 45% of the variance in Self-Esteem (*R*^2^ = 0.45) and 62% of the variance in Perceived Stress (*R*^2^ = 0.62). The detailed results for the direct, interaction, and conditional indirect effects are presented in Table 2. A visual summary is provided in Figure 1.

**Table 2:** Results of the latent moderated mediation analysis (*N* = 425).

Path	*B*	SE	*β*	95% CI
Direct effects				
Predicting self-esteem (mediator)				
Neuroticism → Self-esteem	−0.33	0.04	−0.48^**^	[−0.41, −0.25]
Conscientiousness → Self-esteem	0.22	0.04	0.26^**^	[0.14, 0.30]
Predicting perceived stress (outcome)				
Self-esteem → Perceived stress	−0.68	0.07	−0.53^**^	[−0.82, −0.54]
Interaction effect				
Self-esteem × Hope → Perceived stress	0.11	0.04	0.16^**^	[0.03, 0.19]
Conditional indirect effects on perceived stress via self-esteem				
Indirect effect of neuroticism				
Low hope (−1 SD)	0.22	0.04	0.33^**^	[0.25, 0.42]
Mean hope	0.18	0.03	0.25^**^	[0.19, 0.32]
High hope (+1 SD)	0.12	0.04	0.18^**^	[0.11, 0.26]
Indirect effect of conscientiousness				
Low hope (−1 SD)	−0.15	0.04	−0.18^**^	[−0.26, −0.11]
Mean hope	−0.12	0.03	−0.14^**^	[−0.19, −0.08]
High hope (+1 SD)	−0.08	0.03	−0.10^**^	[−0.16, −0.05]
Variance explained (*R*^2^)				
Self-esteem			0.45^**^	
Perceived stress			0.62^**^	

*B*, unstandarded coefficient; SE, standard error; *β*, standardized coefficient. All models control for age, gender, and income. 95% CIs for indirect effects are bias-corrected bootstrap estimates. ^**^p < 0.01.

The main effects supported the mediation hypothesis. As shown in Table 2, both personality traits were significant predictors of self-esteem. Neuroticism was a strong negative predictor (*β* = −0.48, *p* < 0.001), while Conscientiousness was a moderate positive predictor (*β* = 0.26, *p* < 0.001). In turn, Self-Esteem was a very strong negative predictor of Perceived Stress (*β* = −0.53, *p* < 0.001), controlling for the interaction term.

The critical test of moderation involved the interaction between Self-Esteem and Hope in predicting Perceived Stress. The latent interaction term was significant and positive (*β* = 0.16, *p* < 0.01). Simple slope analysis demonstrated that while the relationship between self-esteem and perceived stress remained negative across all levels of hope, its magnitude was significantly attenuated as hope increased. For individuals with low hope (−1 *SD*), the negative effect of self-esteem on stress was robust (*β* = −0.69, *p* < 0.001). For those with high hope (+1 *SD*), this relationship was significantly flattened (*β* = −0.38, *p* < 0.001), indicating a clear buffering effect.

Finally, we tested the full moderated mediation hypothesis by examining the conditional indirect effects of the personality traits on Perceived Stress (via Self-Esteem) at different levels of Hope. As detailed in the bottom panel of Table 2, the results fully supported our hypotheses. The indirect effect of Neuroticism on Perceived Stress was positive and significant at all levels of Hope, but was significantly buffered at high levels of Hope (*β* = 0.18, 95% CI [0.11, 0.26]) compared to low levels (*β* = 0.33, 95% CI [0.25, 0.42]). The protective indirect effect of Conscientiousness was also significant but showed a substitution pattern; it was strongest for individuals with low hope (Effect = −0.18) and weakest for those with high hope (Effect = −0.10). This suggests that for highly hopeful individuals, the incremental protective role of Conscientiousness in reducing stress via self-esteem is diminished, as hope already provides a primary agentic resource for stress management.

Similarly, the protective indirect effect of Conscientiousness on Perceived Stress was negative and significant at all levels of Hope. This protective effect was also strongest (most negative) for individuals with low hope (Effect = −0.18, 95% CI [−0.26, −0.11]) and weakest for those with high hope (Effect = −0.10, 95% CI [−0.16, −0.05]). These findings confirm that hope serves as a key psychological resource that attenuates the processes through which personality traits affect stress via self-esteem.

### 4.4 Results of the qualitative phase: explaining the buffering effect of Hope

The quantitative analysis established that hope significantly moderates the relationship between self-esteem and perceived stress, such that the negative impact of low self-esteem is attenuated for individuals with high hope. To explore the lived experience behind this statistical buffering effect, we conducted in-depth interviews with 20 adults purposefully selected into two groups: those with low self-esteem but high hope (LSE/HH), and those with low self-esteem and low hope (LSE/LH).

Thematic analysis, focusing on the comparison between these two groups, yielded three core themes that illuminate the psychological mechanisms through which hope transforms the experience of stress for individuals with a fragile sense of self-worth: (1) Temporal Scaffolding, which describes a cognitive re-framing of present stressors against a structured future; (2) Self-Worth Decoupling, the ability to insulate one’s core identity from the outcomes of specific stressful events; and (3) Agentic Pathway Generation, a proactive, problem-solving orientation that counteracts helplessness.

#### 4.4.1 Theme 1: Temporal scaffolding vs. present-focused entrapment

A key difference between the two groups was their cognitive orientation toward time when under stress. The LSE/HH group consistently engaged in what we termed Temporal Scaffolding: they mentally constructed a stable and structured future, which served as an anchor to prevent them from being overwhelmed by present difficulties.

Mr. Wei, a 42-year-old project manager (LSE/HH), articulated this process, explaining, “Yes, this project is a disaster right now, and frankly, I feel like I’m failing.” He paused, then continued, “But I pull up the calendar in my head. I know in 3 months, this phase will be over. I think about the next project and the vacation I have planned after that.” This ability to mentally “zoom out” was a deliberate coping strategy. “The stress is real,” he concluded, “but it’s a point on a line, not the entire line. The future gives the present its proper size.”

Another participant in the LSE/HH group, Ms. Fang, described a similar process when dealing with a conflict with her in-laws. “It feels huge in the moment, like our relationship is broken forever,” she said. “But then I force myself to think about next year’s Spring Festival. We will be sitting together again. We will drink tea. This fight will be a small story by then. Remembering that helps me breathe.”

In stark contrast, the LSE/LH group described a state of Present-Focused Entrapment. For them, a stressful event would collapse their temporal horizons, making the present negative experience feel all-encompassing and permanent. Ms. Zhang, a 35-year-old marketing professional (LSE/LH), described her reaction to negative client feedback: “When my boss told me the client was unhappy, it felt like my whole career was a mistake. I could not think about next week or next month.” The future was not just inaccessible; it was erased. “All I could think was, ‘Right now, I am a failure, and this feeling is never going to end.’ The future just… vanishes. There is only the feeling of being stuck.” This finding directly illuminates the buffering mechanism: hope provides a cognitive tool to contextualize and manage the emotional impact of a stressor by placing it within a broader temporal framework.

#### 4.4.2 Theme 2: Self-worth decoupling vs. stress-as-indictment

Both groups reported low global self-esteem. However, the LSE/HH group demonstrated a remarkable ability to practice Self-Worth Decoupling under stress. Importantly, this was not a reflection of “stable” self-esteem, but rather an active application of hope’s agentic component. By focusing on future goal-attainment (pathways), these individuals were able to cognitively separate the event (“I failed at this task”) from their core identity (“I am a failure”).

Mr. Liu, a 31-year-old software developer (LSE/HH), explained this after a critical code review: “Okay, my code for this module was not good. It was embarrassing. Ten years ago, that would have sent me into a spiral.” He then described a conscious shift in his thinking. “But now I see it as a data point. It means I need to get better at this specific type of algorithm. Because I know I can find another way to succeed (pathways), it’s a skill issue, not a ‘me’ issue. My worth is not tied to this one piece of code.” This decoupling suggests that high hope allows individuals to maintain a “functional” distance from failure that low self-esteem would otherwise make personal.

This ability to compartmentalize was echoed by Ms. Jin (LSE/HH), who was job searching. “Getting a rejection letter still stings, I will not lie,” she admitted. “The thought ‘I’m not good enough’ is right there. But then another thought comes: ‘Okay, maybe I wasn’t the right fit *for that role*. It does not mean I’m unemployable.’ It’s about keeping the problem manageable and not letting it infect everything else.” Conversely, the LSE/LH group consistently interpreted stressful events as a Stress-as-Indictment. For them, a setback was a verdict on their fundamental inadequacy. Ms. Chen, a 48-year-old administrator (LSE/LH) who made a scheduling error, described the experience in deeply personal terms: “It wasn’t just that I made a mistake. It was like a spotlight suddenly turned on, and everyone could see what I already knew: that I am incompetent.” The event became irrefutable proof of her low self-worth. “The mistake is just proof,” she said quietly. “It confirms everything I secretly believe about myself. Every time something like this happens, it just adds another piece of evidence to the pile.” This highlights a critical function of hope: it serves as a cognitive shield, protecting one’s core self-worth from the immediate damage of a specific failure.

#### 4.4.3 Theme 3: Agentic pathway generation vs. perceived helplessness

The final theme differentiated the groups on a behavioral level. The LSE/HH group translated their hope into Agentic Pathway Generation. When faced with a stressor, they immediately began brainstorming and exploring multiple solutions.

Ms. Li, a 39-year-old entrepreneur (LSE/HH) whose business was struggling, exemplified this: “The sales numbers were terrible. My first thought was panic. But after an hour, I sat down with a notebook and started mapping things out.” She described a tangible process of creating options. “‘Okay, Plan A is not working. What is Plan B? We can try a new marketing channel. Plan C? We can pivot the product slightly.’ Just the act of creating these pathways, even if some are long shots, makes me feel back in control. The stress is still there, but now it’s a problem to be solved, not a fate to be suffered.”

In contrast, the LSE/LH group described a pervasive sense of Perceived Helplessness. Their low hope manifested as a cognitive paralysis, an inability to envision any viable pathway forward. Mr. Wang, a 29-year-old junior accountant (LSE/LH), described his reaction to an overwhelming workload: “I just looked at the pile of folders and my mind went blank. I could not even think about where to start. It felt impossible.” This sense of impossibility shut down any problem-solving attempts. “I just sat there, staring at my screen, feeling the dread build up. I ended up just going home and watching TV, trying not to think about it, which of course made it worse the next day.”

Taken together, these three themes provide a rich, multi-faceted explanation for the quantitative finding that hope buffers the impact of low self-esteem on stress. Hope is not mere optimism; it is a complex psychological resource that allows individuals to re-frame stressors in time, protect their core self-worth, and actively generate solutions. It is this suite of cognitive and behavioral tools that explains why two individuals with equally low self-esteem can have profoundly different experiences of stress.

## 5 Discussion

The present study advances understanding of psychological vulnerability and resilience by demonstrating that hope moderates the mediated pathway from Big Five personality traits—specifically neuroticism and conscientiousness—to perceived stress via self-esteem in a diverse sample of Chinese adults. Quantitatively, the latent moderated mediation model confirmed our hypothesis (H1), revealing significant conditional indirect effects where hope resulted in a substantial attenuation of the detrimental impact of neuroticism on stress (indirect effect at high hope: 0.18, 95% CI [0.11, 0.26]; at low hope: 0.33, 95% CI [0.25, 0.42]). Similarly, the protective indirect effect of conscientiousness was weaker at higher hope levels (indirect effect at high hope: −0.10, 95% CI [−0.16, −0.05]; at low hope: −0.18, 95% CI [−0.26, −0.11]). This specific pattern suggests a substitution or ceiling effect; for individuals already possessing high agentic hope, the “agentic labor” typically provided by high conscientiousness becomes less critical for stress management, as hope serves as a functional alternative. The qualitative phase enriched this by elucidating three mechanisms—Temporal Scaffolding, Self-Worth Decoupling, and Agentic Pathway Generation—that explain how hope disrupts the vulnerability cascade, providing a phenomenological lens on the statistical interaction (*β* = 0.16, *p* < 0.01).

The main mediation pathway, where neuroticism negatively predicts self-esteem (*β* = −0.48, *p* < 0.001) and conscientiousness positively predicts it (*β* = 0.26, *p* < 0.001), with self-esteem in turn strongly negatively predicting perceived stress (*β* = −0.53, *p* < 0.001), corroborates the transactional model of stress (Lazarus and Folkman, 1984). Neuroticism’s role as a vulnerability factor, biasing appraisals toward threat and resource inadequacy (Suls and Martin, 2005), erodes self-esteem, consistent with its overlap with negative affectivity and self-criticism (Judge et al., 2002). This low self-esteem then amplifies stress by fostering helplessness in secondary appraisals (Kernis, 2003), aligning with sociometer theory’s view of self-esteem as a gauge of social threat (Leary and Baumeister, 2000). Conscientiousness, conversely, bolsters self-esteem through disciplined goal pursuit (McCrae and Costa, 2008), mitigating stress appraisals. These findings extend prior research by integrating personality into a full mediational chain, highlighting self-esteem as the proximal mechanism translating personality traits into subjective distress.

Hope’s moderating effect on the self-esteem-stress link refines this model, supporting the stress-buffering hypothesis (Cohen and Wills, 1985) for risk factors, while suggesting a substitution framework for protective assets. The positive interaction term indicates that high hope flattens the slope between low self-esteem and high stress (simple slope at high hope: *β* = −0.37, *p* < 0.001; at low hope: *β* = −0.69, *p* < 0.001). This suggests that hope provides a “resource floor” (Hobfoll, 2002) that prevents resource loss spirals, making the protective influence of other traits like conscientiousness less impactful as hope levels increase. Snyder’s (2002) hope theory frames this as pathways and agency thinking counteracting low self-esteem’s cognitive constriction. High-hope individuals’ attenuated vulnerability aligns with evidence of hope’s inverse ties to distress (Alarcon et al., 2013), distinguishing it from optimism by emphasizing agentic, goal-directed processes (Rand, 2018). This interaction is particularly relevant in China’s transforming society, where socioeconomic shifts heighten vulnerability (Liu et al., 2014), and hope may adaptively reframe stressors as challenges (Colla et al., 2022).

The qualitative themes provide a nuanced justification for these quantitative patterns, bridging statistical abstraction with lived experience. Temporal Scaffolding, where high-hope participants anchor current stressors to a structured future, directly maps onto hope’s future-oriented nature (Snyder, 2002), broadening cognitive repertoires per Fredrickson's (2001) broaden-and-build theory. This contrasts with low-hope individuals’ present-focused entrapment, which exacerbates rumination (Nolen-Hoeksema, 2000). Self-Worth Decoupling further elucidates how hope insulates identity. Critically, our data indicate that this decoupling is not a passive state of “self-esteem stability” or “self-compassion,” but an active cognitive bypass driven by pathways thinking. Because high-hope participants were intensely focused on generating the “next move” or alternative routes to their goals, they had less cognitive bandwidth to dwell on present failures as global indictments of self-worth. Low-hope participants’ stress-as-indictment reinforces vulnerability by conflating events with worth (Kernis, 2003), while hope fosters detachment.

Agentic Pathway Generation captures hope’s proactive essence (Snyder et al., 1996), with high-hope individuals brainstorming solutions to counter helplessness (Folkman and Moskowitz, 2004), versus low-hope paralysis that sustains distress cycles (Bolger and Zuckerman, 1995). These mechanisms collectively explain the conditional indirect effects, showing hope’s transdiagnostic utility in Chinese adults, where cultural emphasis on perseverance may amplify its role as a functional substitute for trait-level discipline. Control variables’ minimal influence underscores the model’s robustness, though age and income’s slight ties to stress suggest contextual moderators within the Conservation of Resources framework (Hobfoll, 1989). The mixed-methods integration resolves prior limitations in main-effect studies by unpacking “how” hope disrupts the personality-self-esteem-stress cascade, offering a holistic view of resilience (Seligman and Csikszentmihalyi, 2000).

## 6 Conclusion and implications

This study provides a comprehensive, multi-method understanding of how hope functions as a critical resilience factor within a well-established vulnerability framework. By testing a moderated mediation model, we move beyond asking whether personality and self-esteem influence stress, to understanding for whom this pathway is most potent. The quantitative findings confirm that the deleterious indirect effect of neuroticism—and the protective indirect effect of conscientiousness—on perceived stress via self-esteem is significantly conditional upon an individual’s level of hope. For those with high hope, the toxic link between low self-esteem and stress is substantially weakened, while the relative importance of conscientiousness as a stress-buffer is diminished due to the presence of hope as a primary agentic resource.

Crucially, the subsequent qualitative exploration demystifies this statistical interaction, revealing the lived cognitive and behavioral machinery of hope. The identified mechanisms—Temporal Scaffolding, Self-Worth Decoupling, and Agentic Pathway Generation—illustrate that hope is far more than a vague sense of optimism. The findings demonstrate that hope operates as a proactive cognitive system; by emphasizing the generation of future pathways, hope allows individuals to structurally reframe adversity and protect their core identity from being defined by specific setbacks. Together, these findings offer a nuanced portrait of resilience, showing that even in the context of trait-based vulnerability and fragile self-worth, the cognitive-motivational resource of hope can actively disrupt the cascade toward distress.

This research holds significant implications for both theory and practice. Theoretically, the findings refine Snyder’s hope theory by moving beyond abstract components to show how pathways thinking specifically facilitates identity protection under conditions of low self-worth. The study advances integration across psychological frameworks by demonstrating that diathesis-stress models and positive psychology are not opposing but complementary. Specifically, it shows how a strength-based resource like hope actively modulates the expression of vulnerability traits such as neuroticism, bridging a longstanding conceptual gap. Ultimately, this advances the transactional model of stress by clarifying that low self-esteem leads to high perceived stress primarily when the individual lacks the pathway-driven capacity to generate alternative coping strategies, specifying a key resource that systematically alters secondary appraisal processes.

These theoretical insights translate directly into actionable practical applications. A primary application is the development of more targeted interventions; practitioners can use assessments of self-esteem and hope to identify the highest-risk “low self-esteem/low hope” profile. Building on the specific mechanisms uncovered, training can equip individuals with skills in temporal scaffolding, using guided future visualization to contextualize immediate stressors. Concurrently, self-worth decoupling can be practiced through cognitive-behavioral exercises that help separate global identity from specific failures. Finally, agentic pathway generation can be strengthened through structured problem-solving drills to counter helplessness. In everyday mental health practice, these strategies can be delivered as brief, low-intensity interventions—for example, a 15-min “future mapping” exercise during a routine counseling session, or a self-guided worksheet for patients to generate alternative pathways when facing a setback. Community mental health workers could integrate hope-building questions (e.g., “What is one small step you could take toward a valued goal?”) into regular check-ins, thereby fostering active coping even when self-esteem is low. Beyond clinical settings, these hope-based competencies have clear organizational and educational relevance, encouraging environments that support pathway generation and explicitly separate task performance from personal worth.

## 7 Limitations and suggestions for future research

This study, while robust in its mixed-methods approach, is not without constraints that should frame the interpretation of its findings. The primary limitation stems from the cross-sectional nature of the quantitative data, which, despite sophisticated modeling, prevents firm causal conclusions about the directional relationships within the mediation pathway. We have therefore framed the statistical results as “associations” and “indirect effects” rather than causal effects, and we rely on prior longitudinal evidence (e.g., Kessler et al., 2009; Ebstrup et al., 2011) to support the assumed temporal order. However, without repeated measures, alternative directional models (e.g., perceived stress predicting personality change) cannot be ruled out, and future research should employ longitudinal designs to test the moderated mediation model causally. Furthermore, the exclusive reliance on self-report measures introduces the potential for common-method bias and may not capture more implicit aspects of self-worth or behavioral stress responses. In terms of scope, the generalizability of the identified hope mechanisms beyond the sampled Chinese cultural context remains an open question, as conceptions of self, agency, and the future are deeply culturally embedded. Finally, the qualitative component, though purposefully sampled to illuminate the statistical interaction, was limited in scale and may not have revealed every possible buffering process.

A further limitation concerns potential conceptual overlap among the key constructs. Although we distinguished hope from optimism, self-efficacy, and resilience in Section 2.2, and self-esteem from self-worth in Section 2.1, some degree of empirical overlap is inevitable. For instance, the correlation between self-esteem and hope was *r* = 0.55 in our sample, and the HTMT value (0.64) fell below the conservative threshold of 0.85, supporting discriminant validity. However, moderate shared variance (≈30%) suggests that hope and self-esteem tap related but distinct aspects of positive self-evaluation and goal-directed thinking. Similarly, neuroticism correlated with perceived stress at *r* = 0.65; while our chi-square difference test confirmed that they are separate constructs, residual overlap may inflate the mediated effect. The substitution effect we observed for conscientiousness and hope also raises the possibility of overlapping agency-related variance. Readers should therefore interpret the conditional indirect effects as reflecting the unique contribution of hope above and beyond its shared variance with other resources. Future studies could use bifactor models or residualized change scores to isolate the unique moderating role of hope. Additionally, while we clarified the distinction between self-esteem and self-worth conceptually, the Rosenberg scale conflates them to some degree; alternative measures such as the State Self-Esteem Scale or implicit association tests could provide more fine-grained differentiation.

These limitations directly inform a clear agenda for subsequent research. To establish causality and unpack temporal dynamics, future work should employ longitudinal or experience-sampling designs that track, for instance, how daily shifts in hope moderate the link between self-esteem fluctuations and stress appraisals. Concurrently, investigating the cultural boundaries of these mechanisms is essential; comparative studies could assess whether a strategy like Temporal Scaffolding holds similar salience in cultures with a strong present orientation, or if Self-Worth Decoupling functions differently in collectivist versus individualist settings. To overcome the reliance on self-report, a multi-method approach is warranted, integrating behavioral tasks (e.g., measuring cognitive flexibility as a proxy for pathways thinking), physiological stress indicators, and informant reports to triangulate the core constructs. The most direct application of this study lies in intervention development, calling for randomized controlled trials to test training protocols specifically designed around the three mechanisms against existing therapies, thereby evaluating their unique utility. Finally, research must explore the boundary conditions of hope’s buffering effect, examining whether and how its efficacy diminishes in the face of extreme trauma or chronic, uncontrollable adversity, which would highlight the limits of this resource and the need for complementary supports.

## Data Availability

The data analyzed in this study is subject to the following licenses/restrictions: the datasets generated and analyzed during the current study are not publicly available due to the sensitive nature of the psychological profiles and qualitative interview transcripts but are available from the corresponding author on reasonable request. Requests to access these datasets should be directed to LY, 102750@hrbcu.edu.cn.
